# Mobile radiation shielding device for scatter dose reduction during portable radiography in an orthopedic ward

**DOI:** 10.1371/journal.pone.0362759

**Published:** 2026-11-13

**Authors:** Jongwat Cheewakul, Natee Ina, Suphalak Khamruang Marshall, Sitthichok Chaichulee

**Affiliations:** 1 Department of Biomedical Sciences and Biomedical Engineering, Faculty of Medicine, Prince of Songkla University, Hat Yai, Songkhla, Thailand; 2 Radiological Technology Program, Department of Radiology, Faculty of Medicine, Prince of Songkla University, Hat Yai, Songkhla, Thailand; Universiti Teknologi Malaysia, MALAYSIA

## Abstract

Portable radiography in orthopedic wards exposes healthcare professionals and adjacent patients to scatter radiation in open, shared clinical environments. In crowded wards, maintaining an optimal distance or clearing the area during exposure is often impractical. This study designed, constructed, and evaluated a foldable mobile radiation shielding system comprising two three-panel units, each with a chromed steel frame (210 × 180 cm) with 0.6-mm nominal lead sheets covered by a vinyl outer layer (66 × 100 cm) per panel, mounted on wheeled castors for bedside deployment. The two units were deployed together, one along each lateral side of the bed. Scatter dose rates were measured using an radiation survey meter and a portable X-ray unit during phantom radiography across common orthopedic projections at eight angular positions (0°–315° in 45° increments) and five distances (50–250 cm). The two-unit configuration achieved a median scatter dose reduction of 94.1% (IQR: 88.0%–97.7%; Wilcoxon W = 51,360, *N* = 320, *p* < 0.001). Reductions were broadly consistent across tube voltages (65–85 kVp) and remained substantial across all measurement distances (84.3%–89.6% across 50–250 cm), but varied significantly by angular position, with the highest reduction at 90° (mean 96.91%) and the lowest reductions recorded at 0° (mean 54.02%) and 180° (mean 75.69%). These two directions represented the open axial ends of the setup and were not directly covered by the side-mounted shielding panels. Under a representative high-workload scenario, the two-unit configuration reduced estimated annual occupational doses across all projections, with all post-shielding means remaining well below the average annual occupational dose limit of 20 mSv/year. User feedback was more favorable among radiologic technologists (mean 4.80/5) than among orthopedic ward nurses (mean 3.67/5). The two-unit configuration showed substantial scatter reduction and may serve as an additional protective measure during portable radiography in orthopedic ward settings, with positioning guidance required to maximize protection at clinically relevant angular locations.

## Introduction

Portable X-ray imaging is routinely performed in inpatient wards to support urgent clinical decisions when transporting patients to a fixed radiology suite is unsafe or impractical [1]. Orthopedic wards are among the heaviest users of bedside radiography because many patients have fractures, spinal injuries, post-operative immobilization, traction, external fixators, or limited mobility [1,2]. Although portable imaging improves access to diagnostics, it introduces a persistent radiation protection challenge as examinations are conducted in open, shared spaces where scatter radiation can reach staff, nearby patients, and caregivers [1,3]. Radiation protection programs in hospitals are typically designed around the assumption that diagnostic X-ray exposures are low per examination and that staff work in controlled imaging rooms [4–6]. Portable radiography challenges these assumptions because imaging is relocated into an open environment and may involve multiple staff groups [1,3,7]. Radiologic technologists are usually responsible for positioning the tube and detector, while nurses may remain nearby to monitor patients, manage pain, or maintain immobilization devices. In such settings, even when individual scatter doses are small, repeated examinations can accumulate [2,7].

Scatter radiation arises when primary X-ray photons interact with matter and undergo Compton scattering. These deflected photons are lower in energy than the primary beam but can travel in multiple directions, producing a non-uniform dose field around the bedside [3,8,9]. In a dedicated radiographic room, scatter is mitigated by structural shielding, controlled access, and standardized operator positioning [4–6]. In contrast, orthopedic wards often contain multiple patient beds, narrow walkways, fixed equipment, intravenous poles, monitors, and frequent clinical activity. These constraints can limit the ability of radiologic technologists and nurses to maximize distance, leave the immediate bedside area, or maintain a protected position during every exposure [1,3,7].

Radiation protection in diagnostic radiology is commonly summarized as the principles of time, distance, and shielding. In routine practice, limiting staff presence near the patient at the time of exposure, maximizing distance from the bedside, and maintaining a position outside the main scatter field are highly effective means of reducing occupational dose, although the extent to which these measures can be applied depends on workflow and the physical layout of the clinical environment [1,2,8,9]. Exposure settings should likewise remain appropriate to the clinical task so that output is not higher than necessary. Shielding is therefore particularly important when time and distance cannot be optimized consistently [4–6]. Personal protective equipment (PPE) such as lead aprons may not always be available to all staff in a ward and can be uncomfortable for extended wear, particularly for nurses who may be attending to patients during a procedure [10,11]. Moreover, PPE primarily protects the wearer and does not create a controlled zone for adjacent beds or for transient ward traffic. International and national guidance frameworks emphasize keeping occupational exposure as low as reasonably achievable (ALARA) and maintaining dose limits for radiation workers [4–6]. While the specific regulatory limits and monitoring requirements depend on jurisdiction, the practical implication for ward-based portable imaging is consistent: dose should be reduced where feasible without compromising patient care [4–6]. Because portable imaging can be frequent in orthopedic wards, engineering controls such as mobile shields may provide a systematic reduction that complements, rather than replaces, good radiographic practice and does not rely solely on individual behavior [12–14].

Several real-world factors further amplify the need for ward-based shielding. First, portable radiography may be performed repeatedly on the same patient [2,7,15]. Second, surge conditions such as infectious outbreaks can increase bedside imaging volume because it minimizes patient transport and cross-unit contamination. This has motivated several recent dose-distribution studies during portable examinations [2,3] and during the COVID-19 pandemic [16–18]. Third, orthopedic wards commonly include patients who require close monitoring and careful handling, which can lead to staff remaining in the immediate vicinity of the bed even at the moment of exposure [3,7,19].

Prior studies have characterized scatter distribution during portable and bedside radiography and consistently report that dose rate varies substantially with angle, distance, patient thickness, bed height, and projection [1–3,8]. Importantly, scatter fields are anisotropic. Dose peaks are frequently observed in oblique directions around the bed rather than directly in front of the tube [1,3,8,9]. The scatter field also varies with projection [1,3,8,9]. For example, lateral projections of the lumbosacral (L-S) spine typically require higher output and greater effective thickness, producing higher scatter, whereas extremity projections may generate lower total scatter but still create high local dose rates at short distances [3,8,9]. In addition, the spatial layout of ward rooms and bed arrangements means that a single safe spot is rarely available. A shielding device that is effective in one direction may not be equally effective in another unless it is positioned intentionally. Therefore, a practical shielding strategy in an open ward must protect not only the technologist at the tube but also staff and other patients located in common oblique positions and adjacent-bed locations [1–3,8,9].

Mobile shielding barriers are a promising approach for creating a temporary protected zone in open clinical areas. In interventional environments, mobile shields can reduce dose to staff when positioned correctly [12,20]. The goal of a ward-based shield is not to replace good radiographic practice, but to make safer practice easier to achieve [21,22]. From an engineering perspective, the barrier must balance attenuation with weight and maneuverability [13,14]. Lead sheets provide high attenuation but are heavy. Composite materials may reduce weight but require validation of lead equivalence and durability [13,14]. From a workflow perspective, the shield must be deployable within seconds, must not obstruct access to the patient in an emergency, and must be cleanable with standard hospital disinfectants [12,22].

The present work evaluated a deployable bedside shielding configuration for portable radiography in an orthopedic ward under standardized phantom conditions. Rather than focusing on material attenuation alone, the study examined how shielding performance varied across clinically relevant projections, angular positions, and distances in an open ward geometry. Common bedside projections were assessed using corrected scatter dose rates, percentage reduction, and a representative occupational dose scenario. The aim was to provide a practical geometry-aware assessment of scatter reduction that may inform implementation of temporary shielding barriers during portable imaging in shared ward environments.

The objectives of this study were: (1) to construct a foldable mobile shielding barrier system featuring 0.6-mm nominal lead sheets within the active shielding region for bedside use in an orthopedic ward; (2) to quantify scatter dose reduction across common portable radiographic projections under standardized ward-like geometry; (3) to determine how shielding performance varied according to angular position and distance; (4) to estimate the potential reduction in occupational exposure under a representative high-workload scenario; and (5) to obtain preliminary user feedback from radiologic technologists and orthopedic ward nurses. An early-stage summary of the device design and initial evaluation was presented as a conference abstract at the ISRRT World Congress 2024 [23]. This article provides the first comprehensive report of the study.

## Materials and methods

### Device design and construction

The shielding system was designed as two foldable mobile barriers for bedside radiographic procedures (see Fig 1). The two units were deployed together, one along each lateral side of the bed. They were intended for rapid bedside deployment as a temporary shielding barrier during portable imaging. Because the shielding panels were opaque, the current prototype prioritized attenuation, robustness, and ease of fabrication. Visual contact during imaging could be maintained through the open upper section and the middle section enclosed with transparent vinyl. Each barrier consisted of a chromed steel tube frame with three sections, each 70 cm in width, connected by vertical hinge joints. A lead panel measuring 66 cm in width and 100 cm in height was mounted within each section, forming the active shielding region of the panel. The panels used commercially supplied lead sheets of 0.6 mm nominal thickness, with shielding performance evaluated directly using the assembled system. The lead sheet spans a 100 cm band of the 180 cm panel height, below a 20 cm open upper section. The remaining lower section of each panel was finished in vinyl only, without lead shielding, to reduce weight. This lower section does not provide the same attenuation as the leaded area and may leave seated staff or patients in adjacent low beds with reduced protection. The remaining 4 cm of each section width (2 cm on each lateral edge) consisted of the structural steel frame housing that retained the lead panel. This frame material was not radiologically inert but provided substantially less attenuation than the lead panel itself. The three-panel foldable configuration provided broad side coverage and rapid deployment in a ward setting. This nominal shielding thickness is consistent with IAEA guidance for secondary protective barriers in diagnostic radiology and is comparable to commercial mobile radiation shields used in similar clinical settings [6,13]. The panel surfaces were finished with vinyl sheets for routine cleaning, aesthetic, and clinical use. The device was mounted on rubber-tyred castor wheels with foot-operated locks to maintain stability during imaging. When fully deployed, the three-section configuration provided a total width of 210 cm and an overall height of 180 cm. The device was fabricated in-house at the Faculty of Medicine, Prince of Songkla University. Each assembled unit weighs approximately 30 kg, of which the three lead sheets contribute 13.5 kg and the chromed steel frame about 11 kg. Because two units make up the complete shielding configuration, the total weight of the two-unit system is approximately 60 kg. Because each unit is wheel-mounted, deployment does not require lifting, and a single person can typically position an unfolded unit at the bedside in under one minute.

**Fig 1:**
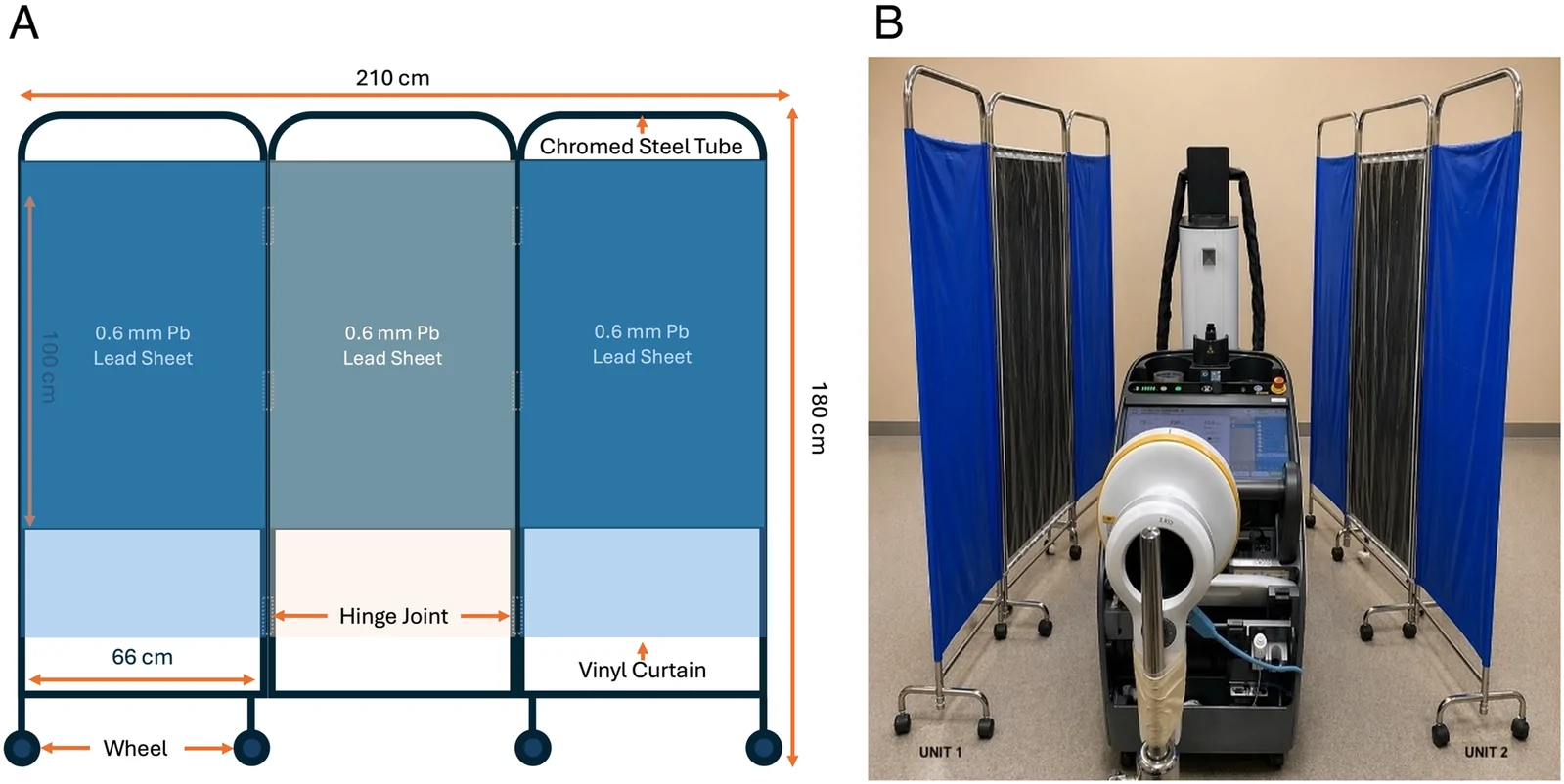
Mobile radiation shielding device. (a) Design schematic of the three-panel foldable shielding barrier mounted on a wheeled chromed steel frame. Each panel incorporated a 0.6-mm nominal lead sheet covered by a vinyl outer layer for surface protection and easy disinfection. When fully deployed, the device measured 210 cm in total width and 180 cm in overall height; the shielding section height was 100 cm and each lead sheet was 66 cm wide. The panels were linked by hinge joints to permit folding and mobility. (b) Photograph of the fabricated shielding devices in the deployed position. Two units of the three-panel device were fabricated; during measurements, one unit was positioned along each lateral side of the bed, as shown in the measurement layout (Fig 3).

### Radiation source, phantoms, and dosimeter

All measurements were performed using a portable X-ray unit (Samsung GM85; Samsung Electronics Co., Seoul, Republic of Korea). The unit’s quality-control test, performed by an ISO/IEC 17025-accredited laboratory, reported a measured filtration of 3.13 mm Al against a criterion of ≥ 2.9 mm Al, a radiation output of 62.0 μGy/mAs, and tube-potential accuracy within 5.9% across 50–110 kVp. Half-value layers at the individual tube voltages used in this study were not separately measured. Scatter radiation was generated using a set of anthropomorphic sectional phantoms (RSD Anthropomorphic Phantom Series; Radiation Support Devices, Inc., Long Beach, CA, USA) representing the thorax, pelvis, femur–leg, and knee regions (see Fig 2). The phantom set represents an average adult male of 175 cm height and 74 kg body weight. Internal structures are fabricated from tissue-equivalent materials. The phantoms are constructed in a transparent polycarbonate-encased configuration, which facilitated visual verification of phantom positioning during each exposure. The phantom components were positioned according to the anatomical region under evaluation for each projection (see Fig 2), so that each exposure represented the relevant body region rather than a simultaneous whole-body assembly of all sections.

**Fig 2:**
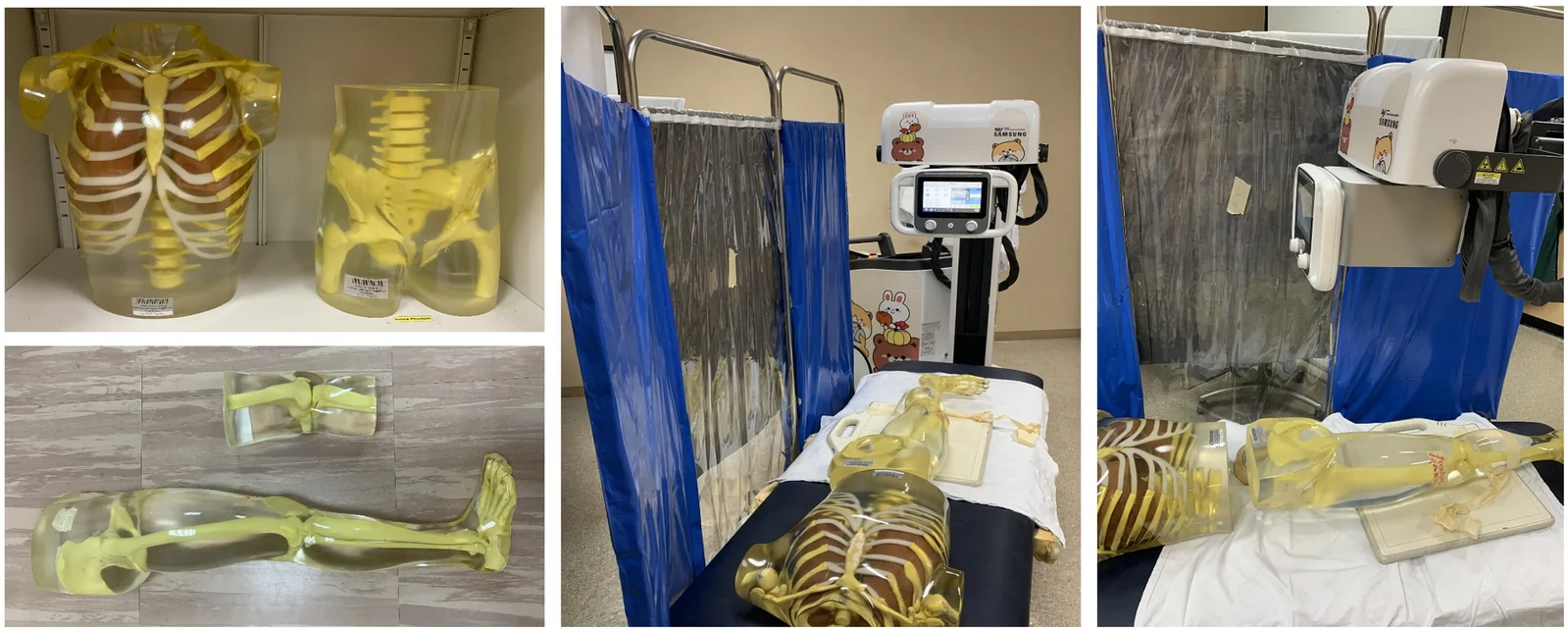
Phantom models and experimental setup during portable radiography. (Left) Transparent RSD anthropomorphic sectional phantoms representing the chest, pelvis, femur-leg, and knee of an average adult male (175 cm, 74 kg). (Center and right) Example images of the experimental setup, showing phantom positioning and the knee lateral projection during portable radiography.

Eight main orthopedic radiographic projections were evaluated at a detector height of 120 cm above floor level: Chest AP Supine, Pelvis AP, L-S Spine AP, L-S Spine Lateral, Femur AP, Femur Lateral, Knee AP, and Knee Lateral (see Table 1). Exposure parameters for each anatomical region and projection were selected to reflect routine portable radiographic practice in the orthopedic ward and are summarized in Table 1. For each projection, the phantom was centered within the irradiation field according to routine clinical positioning for the anatomical region examined, and the bed center was used as the reference origin for angular and radial scatter measurements. Scatter dose was measured using a calibrated ionisation chamber survey meter (RaySafe 452; Unfors RaySafe AB, Billdal, Sweden) operated in dose-rate mode to record ambient dose equivalent, H*(10), in μSv/h. The instrument has an energy response range of 33 keV to 6 MeV and a resolution of 0.001 μSv, which is suitable for diagnostic radiology energy spectra. Although portable radiography uses short exposure times, repeated standardized acquisitions produced a stable mean scatter field for comparative dose-rate measurements under the experimental conditions. Before data acquisition, the survey meter was allowed to stabilise, background radiation was measured and subtracted from all readings, and repeated measurements were obtained under constant exposure conditions. Instrument calibration was traceable to national standards. For each exposure, the survey meter was operated in its standard dose-rate mode with the manufacturer’s default response settings, and the dose-rate reading registered during the exposure was recorded; each measurement condition was assessed with three independent exposures, as described below. A standard source-to-image distance (SID) of 100 cm was used for all projections.

**Table 1:** Radiographic projections and exposure parameters used in the experiment.

Anatomy Region	Radiographic Position	kVp	mA	Time (s)	mAs
Chest	AP (Anteroposterior)	80	320	0.005	1.6
Pelvis	AP	75	250	0.05	12.5
L-S Spine	AP	80	250	0.08	20
L-S Spine	Lateral	85	250	0.2	50
Femur	AP, Lateral	70	200	0.04	8
Knee	AP, Lateral	65	200	0.032	6.4

### Measurement protocol

For each projection, scatter radiation was measured around the patient bed at eight angular positions: 0°, 45°, 90°, 135°, 180°, 225°, 270°, and 315° (see Fig 3). In this layout, the 0° and 180° positions did not represent fully shielded directions. Any reduction observed at these angles should therefore be interpreted as a geometry-dependent partial attenuation effect arising from the relative arrangement of the phantom, portable X-ray unit, and barrier, rather than as complete direct shielding by the lead panels. One three-panel shielding unit was positioned along each lateral side of the bed. Accordingly, the 0° and 180° positions represented the two open axial directions of the setup rather than directions directly covered by the shielding panels. Measurements were taken at five distances from the bed center: 50, 100, 150, 200, and 250 cm. The primary measurement height was 120 cm above the floor, approximating the torso level of standing staff and caregivers. The shielding units were positioned adjacent to the lateral edges of the bed, without a deliberate gap, so that the panels stood between the bed and the surrounding measurement positions (see Fig 2). This bedside placement between the scatter source and adjacent personnel is consistent with the barrier positioning used by Chung et al. [12]. The image receptor was positioned in contact with the phantom on the bed surface, and the tube was positioned according to routine clinical technique for each projection. In addition, two additional measurement conditions were assessed for the L-S spine lateral projection: (i) a lower detector-height setup at 60 cm above floor level at angular positions of 45°, 90°, and 135° to evaluate scatter relevant to adjacent beds and seated positions, and (ii) a hinge-region setup at two hinge locations (H1 and H2, see Fig 1) of the shielding device to evaluate potential leakage at panel junctions. The inclusion of multiple measurement conditions was intentional, as the study was designed to evaluate shielding performance across clinically relevant portable radiographic situations encountered in an orthopedic ward. Because scatter radiation is influenced by projection type, angular position, and distance, restricting the analysis to only a limited number of conditions would not adequately represent routine bedside practice.

**Fig 3:**
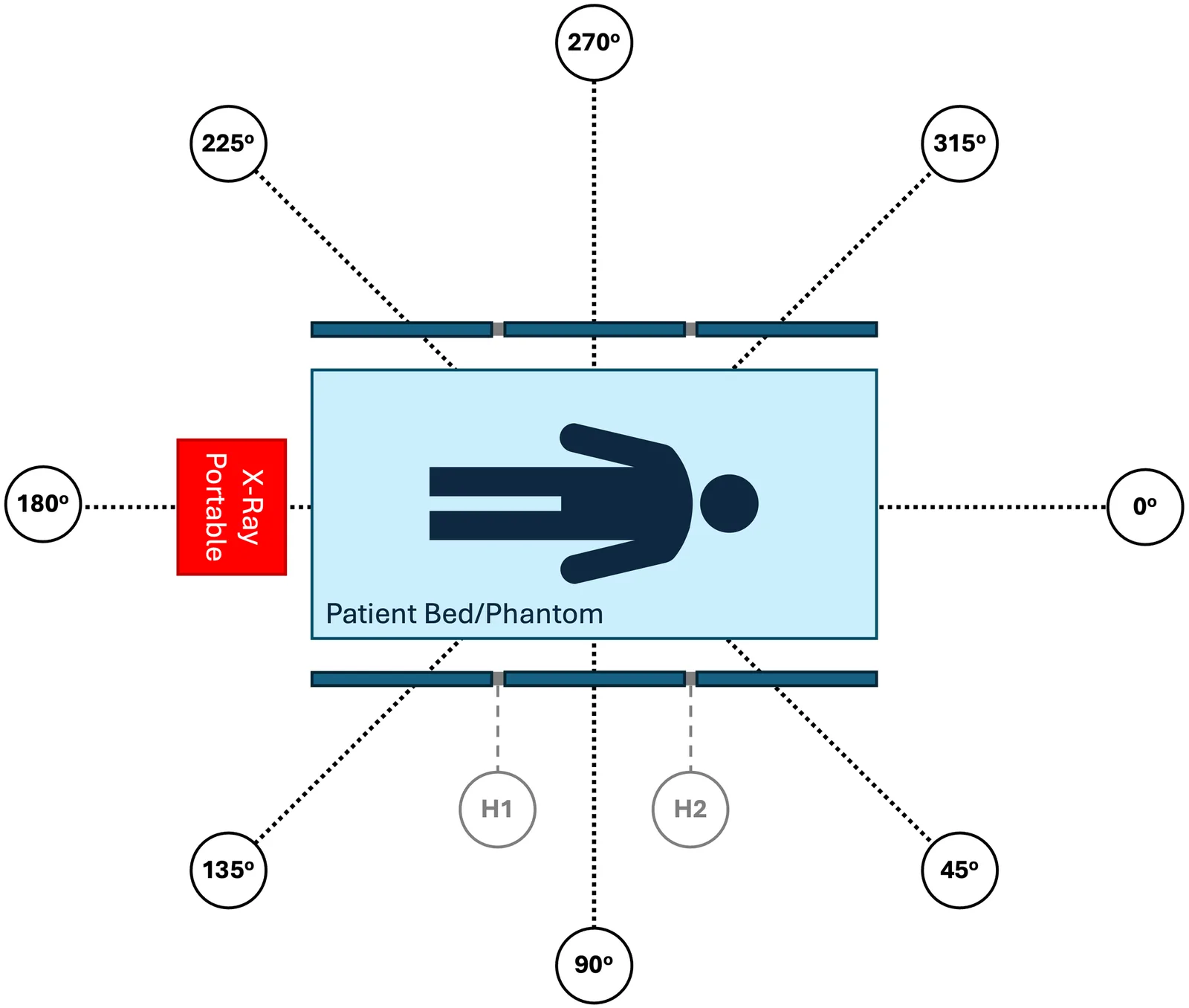
Experimental measurement layout around the patient bed. Measurements include the eight angular positions (0°–315° in 45° increments) and the five measurement distances (50, 100, 150, 200, and 250 cm) from the bed center. Measurement angles were defined relative to the bed center, which served as the reference origin for radial distance measurements. One three-panel shielding unit was positioned along each lateral side of the bed. Accordingly, the 0° and 180° positions represented the two open axial directions of the setup rather than directions directly covered by the shielding panels. H1 and H2 denote the two hinge-region measurement locations.

For each measurement location, the dose rate was recorded in microsieverts per hour (μSv/h) with and without the shielding device. Each measurement condition was assessed three times independently. Within each paired comparison, phantom positioning and exposure parameters were kept constant to isolate the effect of the shielding device. All individual trial readings were retained as recorded. Occasional lower individual readings may reflect the timing of the survey meter’s rate-sampling window relative to the short exposure pulse.

### Dose-rate calculation and shielding performance metrics

Background radiation was measured for five minutes before each session, yielding a mean background radiation dose rate of 0.30 μSv/h. Recorded dose rates were corrected by subtracting the background dose rate. The device calibration certificate reported measured deviations of −1.2% to +4.9% and expanded uncertainties of 5.1%–5.8% (*k* = 2). No additional calibration correction factor was applied to the measured dose rates. Percentage reduction in corrected scatter dose rate was calculated for each paired (projection, distance, angle, repeat) condition as:


$$\begin{array}{c} Reduction\;(\%)=\left[\frac{D_{0}-D_{1}}{D_{0}}\right]\times 100 \end{array}$$

where *D*_0_ is the corrected scatter dose rate without the shielding device and *D*_1_ is the corrected scatter dose rate with the shielding device for the same repeat and measurement condition.

### Occupational dose estimation

Estimated annual occupational radiation dose was calculated from the corrected scatter dose rate using a representative high-workload scenario. For each projection and measurement location, the estimated annual occupational dose (mSv/year) was calculated as:


$$\begin{array}{c} \text{Annual Dose}(\text{mSv/year})=\text{Scatter Dose Rate }(\mu \text{Sv/h})\times \frac{n\times t}{3600}\times d\times 12\times \frac{1}{1000} \end{array}$$

where *n* is the assumed number of portable radiographic exposures per day, *t* is the exposure time per image (s), and *d* is the number of working days per month. Because exposure time varied by radiographic protocol, *t* was assigned according to the corresponding projection-specific exposure parameters shown in Table 1. In the base scenario, *n* was set to 50 exposures/day representing a high-caseload orthopedic ward for a single worker and *d* to 20.83 working days/month (250 working days/year). A sensitivity analysis was additionally performed at *n* = 10 and *n* = 25 exposures/day to illustrate how the estimated annual dose scales with assumed workload. Thus, the estimated annual occupational dose was obtained by scaling the projection-specific daily beam-on time to the assumed monthly and yearly workload. For interpretation, the estimated annual occupational doses were compared with the average occupational dose limit of 20 mSv/year.

Given that the survey meter integrates the dose over the measurement window during short exposure pulses, the registered values represent a time-averaged dose rate. For the purpose of these scenario estimates, these readings were applied as the beam-on dose rate. When combined with the ceiling assumption of the workload, the resulting annual doses should be interpreted as scenario-based estimates when compared to established dose limits.

### User satisfaction survey

User satisfaction was assessed using a structured 10-item questionnaire. Each item was rated on a 5-point Likert scale (1 = very low satisfaction to 5 = very high satisfaction), with an option for not applicable (N/A). N/A responses were excluded on an item-by-item basis, so item-level satisfaction statistics were computed from the available valid ratings for each item and are reported as descriptive summaries.

The questionnaire was administered to 20 radiologic technologists (RT) and 10 orthopedic ward nurses (ON) at the study institution. All radiologic technologists performing portable radiography and all nurses working on the orthopedic ward during the study period were invited, and all responded. Responses were anonymous. No names or identifiers were collected. Respondents rated the device after a hands-on demonstration and trial deployment of the barriers at the bedside in the ward. Some respondents handled the barriers, whereas others, mainly orthopedic nurses, observed the deployment. Respondents were provided an N/A option if they were not involved in each specific question. Informed consent was documented separately from the response forms. As the device was not used during live clinical examinations before the survey, respondents evaluated it on the basis of handling and observation rather than sustained use in clinical workflow.

The questionnaire was self-developed for this study. No pilot testing or formal content-validation procedure was performed. No psychometric properties were assessed. The survey findings serve as preliminary feasibility evidence.

### Statistical analysis

All statistical analyses were performed using paired measurements obtained with and without the shielding device for each measurement configuration. A measurement configuration was defined as a unique combination of radiographic projection, angular position, and measurement distance. The L-S Spine Lateral measurements at 60 cm detector height and at hinge-region locations were reported descriptively and excluded from all primary and secondary statistical analyses. Because measurements were clustered within measurement configuration (three replicate exposures per configuration, with and without shielding), a linear mixed-effects model was adopted as the primary analytical framework, fitted to log-transformed repeat-level corrected scatter dose rates (*N* = 1,920: 320 configurations × 3 replicates × 2 shielding conditions) with measurement configuration included as a random intercept. The overall shielding effect was estimated from a model with shielding condition as the sole fixed effect, and a 95% confidence interval was computed for this effect. To assess whether the shielding effect varied by angular position, measurement distance, and radiographic projection, an extended model additionally included these factors as fixed effects together with their two-way interactions with shielding condition; the significance of each interaction was assessed using likelihood-ratio tests comparing nested models fitted by maximum likelihood. Normality of the percentage reduction in corrected scatter dose rate, and of the log-transformed corrected scatter dose rates used in the mixed-effects and paired-t analyses, was assessed using the Shapiro–Wilk test. As secondary, non-parametric confirmation of the overall shielding effect, given the reduction percentages were not normally distributed, the Wilcoxon signed-rank test was applied to the paired configuration-level corrected scatter dose rates (*N* = 320). As a parametric confirmation, a paired *t*-test was applied to the log-transformed corrected sca*tt*er dose rates, and paired effect size was quantified using Cohen’s $d_{z}$. Because tube voltage (kVp) is a fixed property of each radiographic projection in this design (see Table 1), it is fully nested within projection and could not be modeled as an independent factor once projection was included; the projection term and its interaction with shielding condition are therefore also interpreted as capturing voltage-linked variation. Statistical significance was defined as *p* < 0.05. All raw measurements are provided in the Supplementary Material.

For the user satisfaction survey, item-level satisfaction was summarized using means and standard deviations computed from the available valid responses for each item and is reported descriptively only. For the between-group comparison, one overall satisfaction score was calculated for each participant as the mean of that participant’s valid item ratings, with N/A responses excluded, and the two occupational groups were compared using the Mann–Whitney *U* test on these participant-level scores (20 radiologic technologists versus 10 orthopedic ward nurses). The effect size was expressed as the rank-biserial correlation.

All analyses were performed in Python 3.13.11 using SciPy 1.17.0, statsmodels 0.14.6, and NumPy 2.4.0. Statistical significance was set at α=0.05.

### Ethical approval

This study was approved by the Human Research Ethics Committee of the Faculty of Medicine, Prince of Songkla University (Approval No. REC.66-319-7-2). All methods involving human participants were carried out in accordance with relevant guidelines and regulations and with the Declaration of Helsinki. All participants involved in the user satisfaction survey (radiologic technologists and orthopedic ward nurses) provided informed consent prior to participation. No patients or healthy volunteers were exposed to radiation for the purposes of this research.

## Results

### Overall shielding effect

Our two-unit shielding configuration produced substantial reductions in corrected scatter dose rate across all radiographic projections, angular positions, and measurement distances. The overall median percentage reduction was 94.1% (IQR: 88.0%–97.7%), based on the paired measurement conditions of the eight main projections (see Fig 4). When stratified by shielding geometry, the median reduction was 95.9% (IQR: 92.7%–98.4%) for the six panel-covered directions (*n* = 240 configurations) and 65.7% (IQR: 50.0%–88.3%) for the two open axial directions at 0° and 180° (*n* = 80), which were not directly covered by the panels.

**Fig 4:**
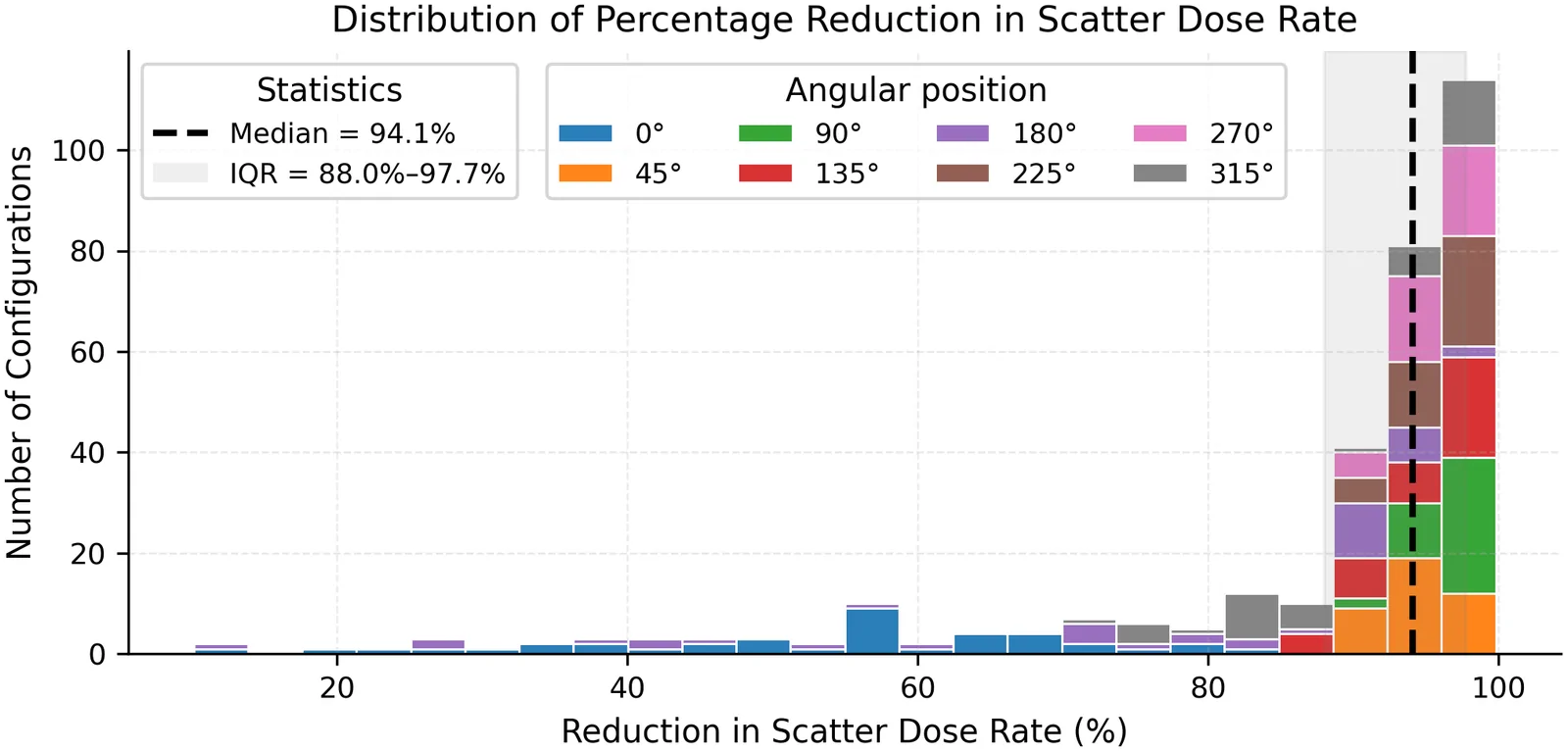
Distribution of percentage reduction in scatter dose rate according to angular position for the main radiographic projections. Dose reduction was generally high across angular positions, with lower reductions observed at 0° and 180°.

As the primary analysis, a linear mixed-effects model fitted to log-transformed repeat-level corrected scatter dose rates, with measurement configuration included as a random intercept, confirmed a highly significant overall shielding effect (β = 2.908, *SE* = 0.035, 95% CI [2.839, 2.977], *z* = 82.628, *p* < 0.001), corresponding to an approximately 18-fold reduction in scatter dose rate with shielding relative to the unshielded condition. As secondary, non-parametric confirmation, reduction percentages were non-normally distributed (Shapiro–Wilk *W* = 0.680, *p* < 0.001; Skewness −2.16, Excess kurtosis 4.25), and the Wilcoxon signed-rank test confirmed a highly significant overall shielding effect (*W* = 51,360, *N* = 320, *p* < 0.001). Parametric confirmation using a paired *t*-test on log-transformed corrected scatter dose ra*t*es yielded the same conclusion (paired *t* = 37.308, *p* < 0.001), with a very large effect size (Cohen’s $d_{z}$ = 2.086). Log-transformed corrected dose rates approached but did no*t* fully satisfy normality by the Shapiro-Wilk test (without shielding: *W* = 0.985, *p* = 0.003; with shielding: *W* = 0.943, *p* < 0.001; paired log-difference: *W* = 0.981, *p* < 0.001). Given the large sample size and effect magnitude, the paired *t*-test is robust to this residual deviation from normality. Mean reductions by projection ranged from 84.13% (L-S Spine Lateral) to 90.01% (Femur Lateral), with an overall mean of 87.22%, averaged across all angular posi*t*ions and projections (see Table 2). Across all the main radiologic projections, the dose-distance relationship was consistently shifted downward by shielding across the full range of distances evaluated (50–250 cm), as illustrated in Fig 5.

**Table 2:** Percentage reduction (%) in corrected scatter dose rate by radiographic projection and angular position.

Projection	Height (cm)	0°	45°	90°	135°	180°	225°	270°	315°	Mean
Chest AP Supine	120	59.79	94.56	96.32	94.02	59.93	95.33	93.98	86.78	85.09
Pelvis AP	120	51.24	95.53	97.86	97.21	80.04	94.58	96.73	88.06	87.66
L-S Spine AP	120	43.94	93.54	95.87	95.99	83.12	94.07	96.18	85.38	86.01
L-S Spine Lateral	120	24.09	94.69	96.10	95.79	84.45	95.53	95.32	87.13	84.13
Femur AP	120	47.54	95.11	97.63	96.33	78.67	97.12	95.96	90.35	87.34
Femur Lateral	120	65.44	96.07	98.02	97.41	81.38	96.00	96.15	89.58	90.01
Knee AP	120	71.18	94.64	97.00	92.36	68.90	97.60	96.34	94.81	89.10
Knee Lateral	120	68.94	94.60	96.52	92.56	69.07	96.53	96.73	92.75	88.46
**Overall Mean**		54.02	94.84	96.91	95.21	75.69	95.85	95.92	89.35	87.22

Values represent percentage reduction in corrected scatter dose rate. Excludes L-S Spine Lateral at 60 cm height and hinge-region conditions.

**Fig 5:**
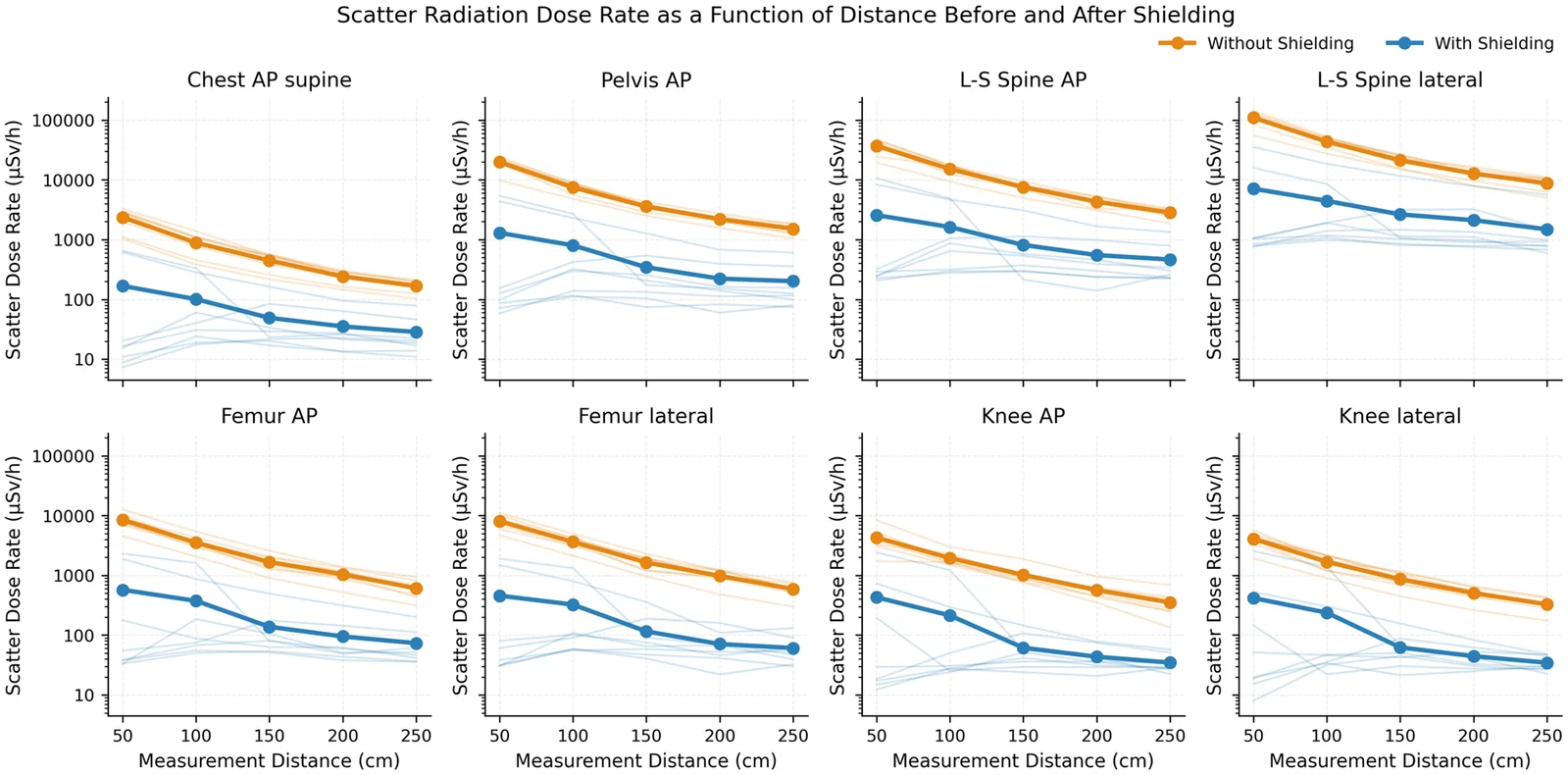
Scatter dose rate as a function of distance with and without the shielding device for the eight main radiographic projections, averaged across all angular positions. In all radiologic projections, shielding shifted the dose-distance relationship downward across the full distance range, with the relative magnitude of this shift greatest at closer distances.

Representative dose-rate values for all measurement configurations are provided in the Supplementary Material to show the absolute magnitude of the unshielded and shielded readings in addition to percentage reduction.

### Spatial pattern of dose reduction

The spatial distribution of shielding performance was non-uniform. Shielding effectiveness varied by angular position. The highest mean reductions were observed at lateral positions (90°: mean 96.91%; 270°: mean 95.92%) and oblique positions (45°: mean 94.84%; 225°: mean 95.85%), where the lead panels were more effectively interposed between the scatter field and the measurement point. Reductions were substantially lower and more variable at 0° (mean 54.02%) and 180° (mean 75.69%). As shown in Fig 6, these two directions corresponded to the open axial ends of the setup and were not directly shielded by the side-mounted lead panels. The reductions observed at 0° and 180° therefore reflect geometry-dependent partial attenuation rather than complete direct shielding by the barrier. This spatial pattern was consistent across all eight radiographic projections (Fig 6a) and across all measurement distances (Fig 6b).

**Fig 6:**
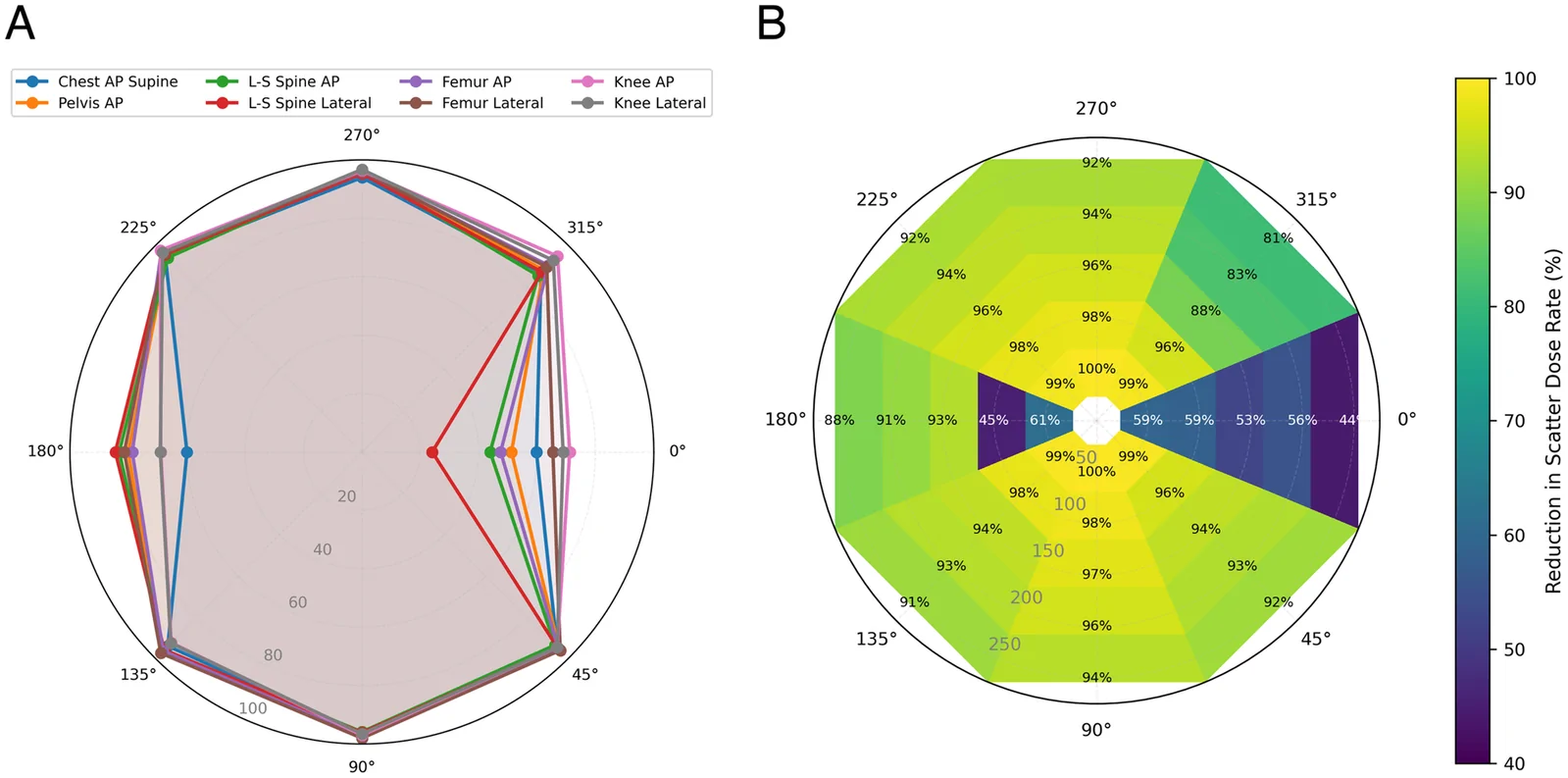
Spatial distribution of mean corrected scatter dose reduction by angular position for the main radiographic projections. (a) Reduction in corrected scatter dose rate across angular positions. Lower and more variable reductions were observed mainly at 0° and 180°. (b) Mean reduction patterns by measurement distance and angular position, persistently high shielding effectiveness was demonstrated across distances with greater variability attributable to angular position.

### Effect of tube voltage, measurement distance, angular position

Mean percentage reductions stratified by tube voltage ranged from 84.13% at 85 kVp to 88.78% at 65 kVp (see Table 3 and Fig 7a). Because these summaries pool data across heterogeneous angular positions, the lower minimum values should be interpreted in the context of direction-dependent shielding performance rather than as stand-alone evidence of random outliers. Because tube voltage is a fixed property of radiographic projection in this design (see Table 1), the shielding × projection interaction from the primary mixed-effects model was examined in place of a separate voltage term. This interaction was statistically significant (likelihood-ratio $\chi^{2}$(7) = 59.81, *p* < 0.001), reflecting the modest range in log-scale attenuation across projections (mean log-ratio 2.70–3.10, corresponding to the 84–90% reduction range already summarized in Table 3). Given the large sample size underlying this test, this statistically significant but small effect is not considered practically important for shielding performance across the tube voltages evaluated. Similarly, mean reductions stratified by measurement distance ranged from 84.26% at 250 cm to 89.56% at 50 cm (see Table 4 and Fig 7b), and the shielding × distance interaction was statistically significant and substantial in magnitude (likelihood-ratio $\chi^{2}$(4) = 977.60, *p* < 0.001). Percentage reduction is a bounded metric and compresses near its ceiling of 100%. On the log scale on which the mixed-effects model was fitted, the mean shielding effect declined from a log-ratio of 4.13 (a 62-fold dose reduction) at 50 cm to 2.18 (a 9-fold reduction) at 250 cm. The device therefore provided substantial protection throughout the distance range evaluated, but its relative attenuation was greatest at close range and diminished with distance, likely reflecting indirect scatter paths around the panel edges at greater distances. Together, these findings indicate that shielding effectiveness, expressed as percentage reduction, remained high across the range of tube voltages and distances evaluated, although the underlying multiplicative attenuation was distance-dependent and, to a lesser extent, projection-dependent. In contrast, angular position had the largest effect on shielding performance among the factors tested (shielding × angle interaction: likelihood-ratio $\chi^{2}$(7) = 1654.87, *p* < 0.001), driven primarily by the lower and more variable reductions observed at 0° and 180°, as described above and illustrated in Fig 7c. From the model including the shielding × angle interaction, the estimated shielding effect was largest at 90° (log-ratio 3.88, 95% CI [3.74, 4.02], a 48-fold reduction) and smallest at 0° (log-ratio 0.85, 95% CI [0.71, 0.99], a 2.3-fold reduction), with 180° intermediate (log-ratio 1.82, 95% CI [1.68, 1.96], a 6-fold reduction).

**Table 3:** Percentage reduction (%) in scatter dose rate stratified by tube voltage.

Tube Voltage	Mean	95% CI	Minimum	Maximum
65 kVp	88.78	[85.30, 92.27]	27.50	99.81
70 kVp	88.67	[85.20, 92.14]	36.00	99.69
75 kVp	87.66	[82.35, 92.96]	42.34	99.76
80 kVp	85.55	[81.41, 89.69]	13.45	99.74
85 kVp	84.13	[76.45, 91.82]	10.16	99.48

Excludes L-S Spine Lateral at 60 cm height and hinge-region conditions. Minimum and maximum values were obtained from pooled observations across multiple angular positions and therefore include geometry-limited conditions.

**Table 4:** Percentage reduction (%) in scatter dose rate stratified by measurement distance.

Measurement Distance	Mean	95% CI	Minimum	Maximum
50 cm	89.56	[84.96, 94.16]	35.99	99.81
100 cm	86.00	[80.54, 91.47]	13.45	99.15
150 cm	88.87	[85.00, 92.74]	22.60	98.43
200 cm	87.43	[83.76, 91.09]	19.11	98.21
250 cm	84.26	[79.95, 88.57]	10.16	95.75

Excludes L-S Spine Lateral at 60 cm height and hinge-region conditions. Minimum and maximum values were obtained from pooled observations across multiple angular positions and therefore include geometry-limited conditions.

**Fig 7:**
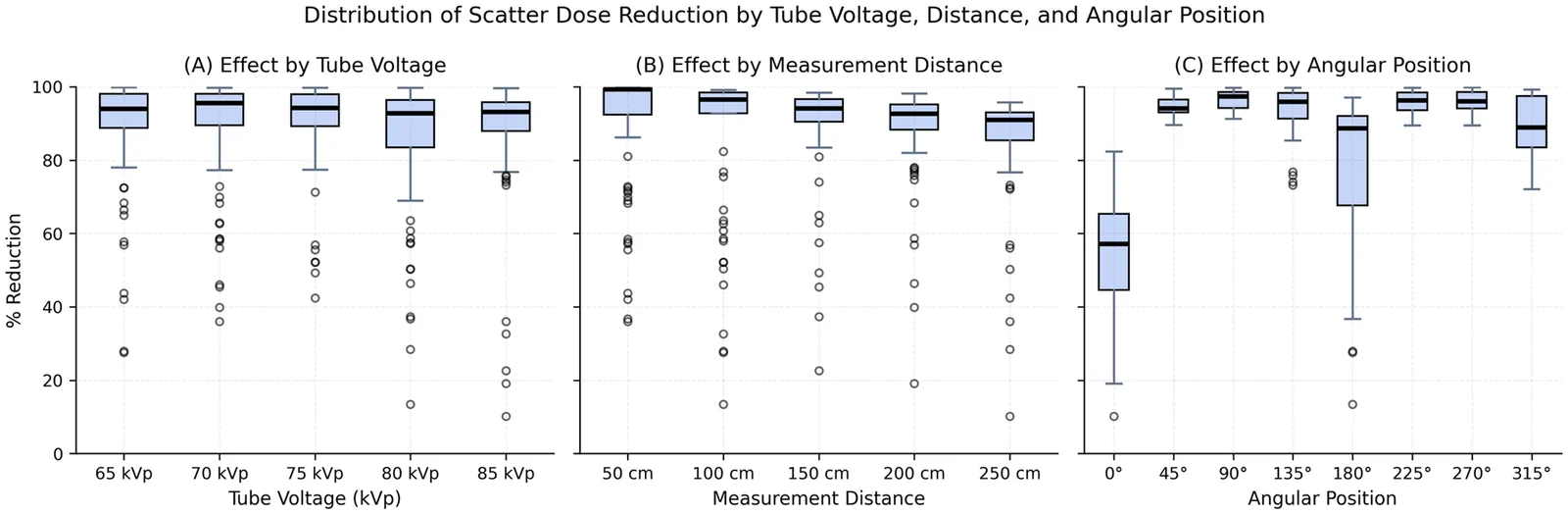
Distribution of scatter dose reduction for the main radiographic projections. Box plots show variation in shielding performance across (A) tube voltage, (B) measurement distance, and (C) angular position. Reduction remained high across tube voltage and measurement distance, whereas angular position showed variations at 0° and 180°.

### Additional measurement conditions

For the L-S Spine Lateral projection evaluated at a detector height of 60 cm, mean reductions across the three measured angular positions (45°, 90°, and 135°) were 93.75%, 92.59%, and 77.19%, respectively, with an overall mean of 87.84%. While substantial reduction was observed at these three 60 cm locations, the lower section of the panels lacks lead shielding; therefore, these results characterize protection at specific angles, and comprehensive height-dependent mapping is required for a full assessment of the lower region.

Scatter dose rates were also measured at the two hinge junctions of the shielding device during the L-S Spine Lateral projection at 120 cm height across distances of 50–250 cm. At Hinge 1 (H1), reductions ranged from 89.01% to 93.17% (mean 91.3%). At Hinge 2 (H2), reductions ranged from 94.75% to 99.60% (mean 97.2%). The higher attenuation at Hinge 2 likely reflects a more favorable geometric relationship between that junction and the primary scatter field. Although both hinge locations produced substantial reductions, the lower performance at Hinge 1 relative to solid-panel lateral positions (typically >93%) suggests a modest degree of leakage at panel junctions, identifying hinge geometry as a potential target for design refinement in future iterations.

### Estimated annual occupational dose

Under the representative upper-bound high-workload scenario (50 exposures/day, 250 working days/year), estimated annual occupational doses without shielding varied substantially by projection, reflecting differences in exposure parameters. The highest mean annual dose without shielding was observed for L-S Spine Lateral at 60 cm (32.13 mSv/year), followed by L-S Spine Lateral at Hinge (31.78 mSv/year), L-S Spine Lateral (27.22 mSv/year), and L-S Spine AP (3.72 mSv/year). Chest AP Supine produced the lowest mean annual dose without shielding (0.014 mSv/year), consistent with its brief exposure time. With shielding, all projection-specific mean annual doses were substantially reduced. The highest post-shielding mean annual dose remained in L-S Spine Lateral at 60 cm (3.36 mSv/year), whereas the greatest relative reduction was observed for L-S Spine Lateral at Hinge (95.70%). All other post-shielding mean annual doses were below 3 mSv/year, with most below 0.5 mSv/year (see Table 5). Across all projections, post-shielding mean annual dose estimates remained well below the average occupational dose limit of 20 mSv/year, as illustrated in Fig 8.

**Table 5:** Scenario estimates based on instrument-reported dose-rate readings of annual occupational dose with and without shielding for all measurement conditions.

Radiologic Projection	Mean Annual Dose (mSv/y)		Reduction
	Without Shielding	With Shielding	
Chest AP Supine	0.0142	0.0013	90.69%
Pelvis AP	1.1995	0.0995	91.70%
L-S Spine AP	3.7153	0.3345	91.00%
L-S Spine Lateral	27.2152	2.4697	90.93%
L-S Spine Lateral at 60 cm	32.1307	3.3645	89.53%
L-S Spine Lateral at Hinge	31.7832	1.3681	95.70%
Femur AP	0.4259	0.0348	91.83%
Femur Lateral	0.4131	0.0286	93.09%
Knee AP	0.1816	0.0174	90.43%
Knee Lateral	0.1659	0.0177	89.35%

**Fig 8:**
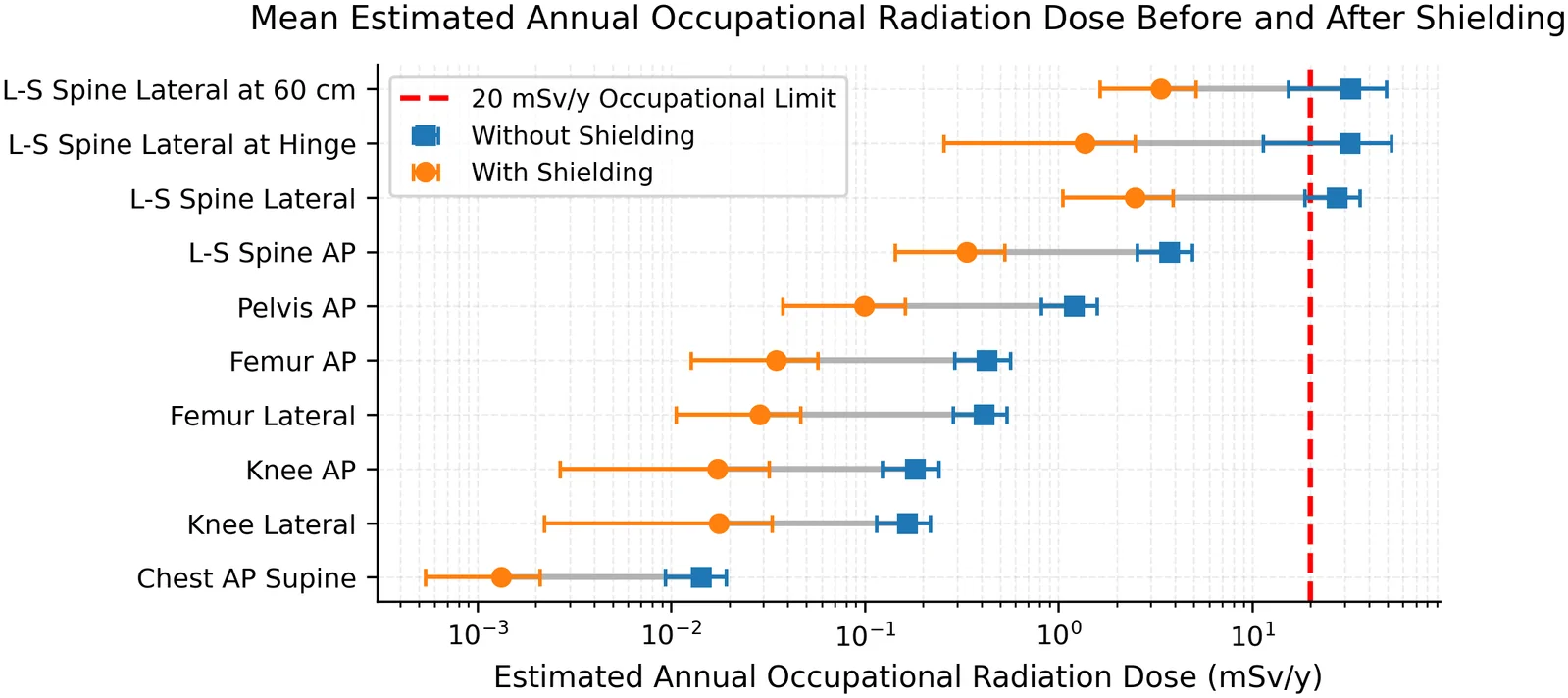
Estimated annual occupational radiation dose with and without the shielding device for all measurement conditions. Points represent means, error bars indicate 95% CIs, and the red line indicates the average occupational dose limit of 20 mSv/year. Shielding reduced the estimated dose in every condition, and all values with shielding were below the limit.

Because the base-scenario workload of 50 exposures/day is a modeled assumption, a sensitivity analysis was performed at 10 and 25 exposures/day (see Table 6). Only the L-S Spine Lateral projection under the 50 exposures/day scenario exceeded the average 20 mSv/year occupational dose limit without shielding (27.22 mSv/year); at 25 exposures/day the unshielded estimate for this projection fell to 13.61 mSv/year, and at 10 exposures/day to 5.44 mSv/year, both below the limit. All shielded estimates remained well below 20 mSv/year at every workload level tested.

**Table 6:** Sensitivity of the scenario estimates based on instrument-reported dose-rate readings of annual occupational dose (mSv/year) to assumed daily workload (10, 25, and 50 exposures/day), without and with shielding.

Projection	10/day		25/day		50/day	
	Without	With	Without	With	Without	With
Chest AP Supine	0.0028	0.0003	0.0071	0.0007	0.0142	0.0013
Pelvis AP	0.2399	0.0199	0.5998	0.0498	1.1995	0.0995
L-S Spine AP	0.7431	0.0669	1.8577	0.1672	3.7153	0.3345
L-S Spine Lateral	5.4430	0.4939	13.6076	1.2348	27.2152	2.4697
Femur AP	0.0852	0.0070	0.2129	0.0174	0.4259	0.0348
Femur Lateral	0.0826	0.0057	0.2065	0.0143	0.4131	0.0286
Knee AP	0.0363	0.0035	0.0908	0.0087	0.1816	0.0174
Knee Lateral	0.0332	0.0035	0.0830	0.0088	0.1659	0.0177

Values computed from the same background-subtracted scatter dose rates and formula as Table 5, varying only the assumed exposures/day. Excludes L-S Spine Lateral at 60 cm height and hinge-region conditions.

### User satisfaction

Participant-level satisfaction scores, each calculated as the mean of that respondent’s valid item ratings, averaged 4.43 (SD 0.62) across both occupational groups on a 5-point Likert scale. Radiologic technologists reported high satisfaction (mean 4.80, SD 0.24; median 4.95, IQR 4.67–5.00; n = 20), and orthopedic ward nurses reported somewhat lower but still favorable ratings (mean 3.67, SD 0.39; median 3.63, IQR 3.51–3.87; n = 10). The difference between groups was significant on a Mann-Whitney U test of the participant-level scores (U = 199.0, p < 0.001), with a rank-biserial correlation of 0.99. Participant scores ranged from 4.20 to 5.00 among radiologic technologists and from 3.12 to 4.33 among nurses. All 20 radiologic technologists rated all ten items, whereas nurses provided between five and ten valid item ratings each.

The highest-rated items across both groups were “The device effectively reduces surrounding scatter radiation” (overall mean 4.80) and “The production cost of the device is reasonable” (overall mean 4.73). Among nurses, the lowest-rated items were physical design and appearance (mean 3.30) and ease of storage (mean 3.50), suggesting that device compactness and form factor may benefit from refinement for routine ward integration. Several items also were given N/A responses among orthopedic nurses, notably “The production cost of the device is reasonable” (2/10 valid responses) and “The device effectively reduces surrounding scatter radiation” and “The device helps reduce radiation exposure to adjacent patients” (5/10 valid responses each). Radiologic technologists rated all items at or above 4.55, with the highest scores for radiation safety perception and hazard concern reduction (both 4.95). Full item-level results are summarized in Table 7.

**Table 7:** User satisfaction ratings for the shielding device by occupational group (5-point Likert scale).

Criterion	RT			ON			Overall		
	N	Mean	SD	N	Mean	SD	N	Mean	SD
The device effectively reduces surrounding scatter radiation.	20	4.95	0.22	5	4.20	0.45	25	4.80	0.41
The device is easy and convenient to use.	20	4.85	0.37	9	3.78	0.44	29	4.52	0.63
The device reduces concern about radiation-related hazards.	20	4.95	0.22	8	3.75	0.71	28	4.61	0.69
I would like to use this device in my workplace.	20	4.80	0.41	10	3.70	0.48	30	4.43	0.68
I am satisfied with the device’s design and appearance.	20	4.55	0.60	10	3.30	0.48	30	4.13	0.82
The device is easy to store, space-saving, and practical.	20	4.80	0.41	10	3.50	0.53	30	4.37	0.76
The device is safe to use.	20	4.80	0.41	9	3.67	0.71	29	4.45	0.74
The device helps reduce radiation exposure to adjacent patients.	20	4.85	0.37	5	4.00	0.71	25	4.68	0.56
The production cost of the device is reasonable.	20	4.80	0.41	2	4.00	0.00	22	4.73	0.46
The device is lightweight and easy to install.	20	4.70	0.47	9	3.56	0.53	29	4.34	0.72
**Score**	**20**	4.80	0.24	**10**	3.67	0.39	**30**	4.43	0.62

RT: Radiologic Technologists (20 respondents); ON: Orthopedic Nurses (10 respondents). *N* denotes valid responses per item. The between-group comparison was performed on these participant-level scores.

## Discussion

### Principal findings

This study demonstrated that a two-unit configuration of purpose-built mobile radiation shielding barriers with 0.6-mm nominal lead sheets substantially reduced scatter radiation during portable radiography across a range of common orthopedic projections. The overall radiation reduction was statistically significant and associated with a very large effect size. Reduction remained high across the tube voltage (65–85 kVp) and measurement distance (50–250 cm) strata evaluated, with only a modest, statistically significant effect of voltage/projection; distance showed a statistically significant and larger effect on the underlying multiplicative attenuation, although percentage reduction itself remained substantial throughout the distance range tested (see Results). The device performs reliably across the range of exposure conditions encountered in routine orthopedic ward practice, with relative attenuation efficiency somewhat greater at closer range. The principal dimension of variability was angular position, with meaningfully lower reductions at 0° and 180°, which represented the open axial directions of the setup rather than directly shielded panel-covered positions. Occupational dose estimates indicated lower projected annual doses across all evaluated scenarios under the modeled workload [4]. Preliminary user feedback was more favorable among radiologic technologists than among orthopedic ward nurses. Taken together, these findings indicate substantial scatter reduction under the standardized phantom-based conditions evaluated in this study and support further refinement of bedside shielding strategies for open ward environments. These findings are consistent with prior evaluations of mobile lead shields in clinical settings [12,13].

### Shielding performance variations

The angular dependence of shielding effectiveness reflects the geometric relationship between the scatter source, the lead panel, and the measurement location. At lateral and oblique positions (90°, 270°, 45°, 225°), the three-panel configuration reliably interposed the lead sheets in the direct path of scattered photons, yielding mean reductions consistently above 93%. At 180°, the measurement point was on the same side as the X-ray tube (see Fig 3), where the tube housing and its cabling likely provided a degree of incidental attenuation in addition to the partial geometric interception described above (mean 75.69%). At 0°, the measurement point was at the fully open end of the setup, opposite the tube, with no equipment in the scatter path, and this position showed the lowest reduction of all eight positions (mean 54.02%). This pattern is consistent with prior characterizations of the anisotropic scatter field around portable radiographic setups, which similarly report oblique-direction dose peaks and direction-dependent variability [1–3,9]. These findings indicate that positioning guidance, i.e., directing staff to lateral or oblique positions relative to the primary beam, should accompany routine deployment of the barrier [21,22].

The lower reductions observed at 0° and 180° should not be interpreted as contradictory shielding performance. In the present layout, both directions represented open axial positions rather than panel-covered directions. Consequently, dose reduction remained measurable but lower than at the lateral and oblique positions, where the lead panels more effectively intercepted the scatter field.

For the hinge-region assessment of the L-S Spine Lateral projection, measurements at panel junctions confirmed the presence of localized leakage, as expected for multi-panel foldable designs. While hinge-region dose rates remained substantially lower than unshielded values at most locations, this finding identifies a potential optimization target for future iterations, such as overlapping panel edges or flexible lead inserts at junctions [13,14].

### Comparison with other mobile shielding devices

The contribution of the present study lies in the structured evaluation of a deployable side-mounted bedside barrier across clinically relevant portable radiography geometries in an orthopedic ward context. The present device achieved a median scatter dose reduction of 94.1% (IQR: 88.0%–97.7%) and an overall mean of 87.22%, comparing favourably with published outcomes from mobile and area shielding devices evaluated in other clinical settings. Scott et al. [13] evaluated the RAMPART M1128 floor-mounted mobile lead shield in a cardiac catheterisation laboratory using clinical dosimetry and demonstrated a 60% dose reduction for the primary operator, with reductions of 65%–84% for other laboratory staff; phantom measurements at the primary operator position yielded 65% reduction at eye level and 90% at chest level. Laish-Farkash et al. [24] evaluated the robotic Radiaction Shielding System (RSS) in bench tests and preliminary clinical use in an interventional cardiology laboratory, demonstrating significant scatter reductions across all personnel positions around the C-arm. Bhindi et al. [25], in a prospective controlled evaluation of the same device during diagnostic and interventional cardiology procedures, reported an overall reduction of 74.7% across all sensor locations and 82.9% for the main physician. Koenig et al. [14] demonstrated a reduction of up to 80.6% using a radiation-absorbing pad at simulated operator heights of 100–165 cm during a transfemoral angiography phantom setup.

The higher overall reductions achieved by the present device likely reflect three factors. First, two three-panel full-height units (each 210 × 180 cm) provide a substantially larger protected area than a single unidirectional screen or an underbed pad. Second, a 0.6-mm nominal lead sheet is consistent with the standard specification for secondary protective barriers in diagnostic radiology and provides substantial attenuation at the energies tested (65–85 kVp). Third, deployment as a bilateral area barrier, one unit on each side of the bed, intercepts scatter from multiple directions simultaneously rather than protecting only the primary operator position.

### Occupational safety implications

The occupational dose estimates highlight that the highest-risk projections in orthopedic wards are those requiring the greatest mAs, particularly L-S Spine Lateral. Elevated scatter exposure during high-output orthopedic and fluoroscopically-guided procedures has been previously documented in orthopedic theatre and ward settings [19,26,27]. Under the assumed high-workload scenario, the mean annual dose without shielding for L-S Spine Lateral exceeded the average 20 mSv/year occupational limit [4], illustrating that cumulative scatter exposure over a sustained high-volume portable radiography workload is non-negligible [2,7,19]. The comparison with this limit should also be interpreted in context for ward nurses, adjacent patients, and caregivers, who may not be classified or monitored as radiation workers and to whom occupational dose limits may not formally apply. With shielding, L-S Spine Lateral fell to 2.47 mSv/year, substantially below the average occupational limit. While the absolute dose contributions of other projections were smaller, their combined effect in a mixed-caseload ward may be additive across an annual workload, though quantifying this combined effect would require a projection-level workload case-mix that was not collected in this study.

The ALARA principle requires that exposure be kept as low as reasonably achievable, not merely below regulatory thresholds [4,5,28]. The consistent and large reductions achieved across all projections support deployment of mobile shielding as a systematic engineering control rather than an optional supplement [12–14]. Importantly, because the two-unit configuration reduces scatter throughout the ward environment rather than only for the device operator, it also attenuates exposure to adjacent patients, nursing staff, and caregivers who may not wear personal protective equipment during bedside radiography [3,10,11].

### Usability and implementation potential

User feedback indicated a difference between occupational groups. Radiologic technologists rated the device more favorably, whereas orthopedic ward nurses expressed greater concern about compactness, storage, and physical integration in a constrained ward environment. The gap between groups may reflect differences in the nature of interaction with the device. Radiologic technologists deploy and position the shield as part of their procedural workflow, while nurses encounter it primarily as an additional object in an already-constrained ward environment. Studies have consistently reported lower radiation protection awareness and compliance with protective equipment among non-radiology nursing staff [11,29–31], which may partly explain the more cautious satisfaction ratings observed in this group. Nurses rated physical design and storage dimensions somewhat lower than technologists, suggesting that future versions could benefit from a reduced folded footprint, lighter frame materials, or a more streamlined profile compatible with narrow ward bay layouts. Although the castor mounting means each unit is wheeled rather than lifted, navigating two 30 kg units within a confined ward bay may have contributed to the more moderate nurse rating for the “lightweight and easy to install” item.

The shielding device incorporates several design features that address recognized practical barriers to consistent barrier use in clinical settings [22]. Rapid deployment via the hinged three-panel configuration, wheel-mounted mobility with foot-operated locks, and a vinyl outer surface compatible with standard disinfection protocols all support routine integration into portable radiography workflows. These features are aligned with the principle that engineering controls succeed in ward environments only when they are practical and reliable enough to be used consistently without adding significant procedural burden [12,22].

### Practical positioning guidance

Based on the direction-dependent reduction patterns observed in this study, the following recommendations are proposed for clinical implementation. First, the barrier should be placed as close as feasible to the patient bed on the side where staff are expected to stand. Mean reductions were consistently high across all measurement distances, and the barrier remained effective throughout the full distance range tested; however, because relative attenuation efficiency was greatest at close range and diminished with distance (see Results), closer placement is preferred both to minimise angular gaps at panel edges and to maximise attenuation performance. Second, during exposure, staff should stand fully behind the barrier panels in a lateral or oblique position relative to the primary beam. Third, barrier deployment may be considered as an additional protective step in portable radiography workflows when bedside conditions limit optimization of distance and staff positioning. This is consistent with the principle that engineering controls are most effective when integrated systematically into clinical routines [12,22].

### Strengths and limitations

This study has several strengths. Measurements were conducted under standardized phantom conditions replicating routine portable radiographic practice across eight common orthopedic projections. The spatial sampling grid—eight angular positions and five distances per projection—provides comprehensive characterization of shielding performance. Paired measurement configurations with three replicates per condition, analyzed using a repeated-measures-aware mixed-effects model as the primary framework, with nonparametric and parametric methods as secondary confirmation, yield robust and internally consistent evidence. The occupational dose scenario translates physical dosimetry findings into a clinically relevant reference frame [4].

Several limitations are acknowledged. Because the current prototype used opaque shielding panels, it may be less suitable in situations requiring uninterrupted full visual observation of the patient during exposure. Future designs should evaluate transparent shielding materials, observation windows, or hybrid panel configurations. Phantom measurements, while reproducible and controlled, do not fully replicate the scatter environment of a real ward, where bed geometry, curtain partitions, additional equipment, and reflective wall surfaces may modify the scatter field [1,9]. All measurements used a single anthropomorphic phantom set representing an average adult male (175 cm, 74 kg). As scatter dose rate is known to vary with patient thickness and body habitus, the reduction percentages reported here may not generalize directly to substantially smaller or larger patients, or to other gender and age groups. This should be considered when extrapolating the findings to individual patients outside this reference body size. Although only three repeated measurements were performed per condition, the observed standard deviations were consistently small, suggesting stable and repeatable measurements under the controlled phantom-based setup. Reporting statistics from triplicate measurements is common in experimental radiological studies. Nevertheless, a larger number of repeats and a formal uncertainty budget would strengthen the dosimetric measurements. Real occupational exposure patterns depend on task assignment, shift structure, and worker position variability, which are not captured by the fixed-position scenario modeled here [7,26]. The occupational dose estimates in Tables 5 and 6 are modeled per-projection ceilings, each assuming the stated number of exposures/day of that single projection type. These estimates are not intended to be summed across projections, as an individual worker’s annual dose would depend on the actual case mix of examinations performed. Because projection-specific workload data were not available in this study, a cumulative per-worker annual dose was not estimated. The annual occupational dose values were extrapolated from measured scatter dose rates under specified exposure scenarios. The dose-rate meter response during short exposure pulses was not separately assessed. These values should therefore be interpreted as scenario-based estimates rather than direct occupational dose measurements. The user satisfaction survey was conducted at a single institution with a limited sample, particularly for orthopedic nurses (n = 10), and findings may not generalize to wards with different layouts or staffing configurations [29,31]. Comprehensive angular and height mapping beyond the primary 120 cm measurement plane was beyond the scope of this study. Lower-height conditions were evaluated only for the L-S Spine Lateral projection at three angular positions. Finally, long-term durability of the lead sheets, frame, and wheel mechanism under routine clinical use was not assessed and should be evaluated in future work [13].

## Conclusion

Two foldable mobile shielding units with 0.6-mm nominal lead sheets, deployed one on each side of the bed, produced substantial reductions in scatter radiation under the standardized phantom-based conditions evaluated in this study. The median reduction was 94.1%, although shielding performance varied significantly by angular position and was lower in the open axial directions of the setup. Estimated occupational dose was reduced in all evaluated projection scenarios, with all post-shielding mean values remaining below the average 20 mSv/year occupational limit in the modeled workload. Preliminary user feedback was more favorable among radiologic technologists than among orthopedic ward nurses, with nurse responses indicating the need for further refinement in compactness, storage, and ward integration. These findings support the use of temporary bedside shielding as an additional protective measure in portable orthopedic ward radiography, while also indicating the importance of positioning guidance, design refinement, and further evaluation under real clinical workflow conditions, including personal dosimetry validation, before the observed reductions can be assumed to translate directly into reduced clinical occupational dose.

## Supporting information

S1 File Raw experimental scatter dose-rate measurements. Trial-level scatter dose rates recorded with and without the shielding device for all measurement configurations, provided as a spreadsheet with one sheet per condition: the eight main radiographic projections at 120 cm detector height, the L-S Spine Lateral projection at 60 cm detector height, and the L-S Spine Lateral hinge-region measurements at 120 cm. Each sheet reports raw and background-corrected dose rates, percentage reduction, and estimated annual occupational dose for every angular position, distance, and repeat measurement.
(XLSX)

S2 File User satisfaction survey response counts. Number of responses at each rating (5–1) and N/A for each of the ten questionnaire items, with valid N, mean, and SD, by occupational group (radiologic technologists, n = 20; orthopedic ward nurses, n = 10).
(XLSX)
